# Supplementary material for: Metabolic Profiling for Unveiling Mechanisms of Kushenol F against Imiquimod-Induced Psoriasis with UHPLC/MS Analysis
Source: Molecules. 2024 May 21;29(11):2410. doi: 10.3390/molecules29112410 (PMC11173924; doi:10.3390/molecules29112410)
Supplement: Supplementary file 1 [file molecules-29-02410-s001.zip › molecules-2943100-supplementary.pdf]

Supplementary Materials

Metabolic Profiling for Unveiling Mechanisms of Kushenol F against Imiquimod-Induced Psoriasis with UHPLC/MS Analysis

Zhen Chen [1,†], Jiaoli Cheng [1,†], Xunqing Yin [2], Ting Ao [2], Xudong He [2], Yaqin Yang [3,*], Yuping Lin [2,*] and Xingxin Yang [2,*]

1 Science and Technology Achievement Incubation Center, Kunming Medical University, Kunming 650500, China; chenzhen@kmmu.edu.cn (Z.C.); jiaoli_cheng980217@163.com (J.C.)
2 College of Pharmaceutical Science, Yunnan University of Chinese Medicine, Kunming 650500, China; yinxunqing1018@163.com (X.Y.); ace153790@163.com (T.A.); hexudong@ynutcm.edu.cn (X.H.)
3 School of Pharmaceutical Sciences, Zhejiang Chinese Medical University, Hangzhou 311403, China
* Correspondence: rc.yaqin0902@zcmu.edu.cn (Y.Y.); linyuping1221@163.com (Y.L.); yxx78945@163.com (X.Y.); Tel.: +86-571-86633177 (Y.Y.); +86-871-65933303 (Y.L. & X.Y.)
† These authors contributed equally to this work.

Table of Contents

Supplementary figures

1. Representative total ion current profiles of the skin samples in the negative and positive ion mode.

2. The chemical structures of 161 potential biomarkers.

1 Supplementary figures

Figure S1. Representative total ion current profiles of the skin samples in the negative and positive ion mode. Significant differences were observed in peak number and intensity between the four groups, indicating different metabolomic states in the different groups. A, control group; B, model group; C, KSCF group; I, positive ion mode; II, negative ion mode.

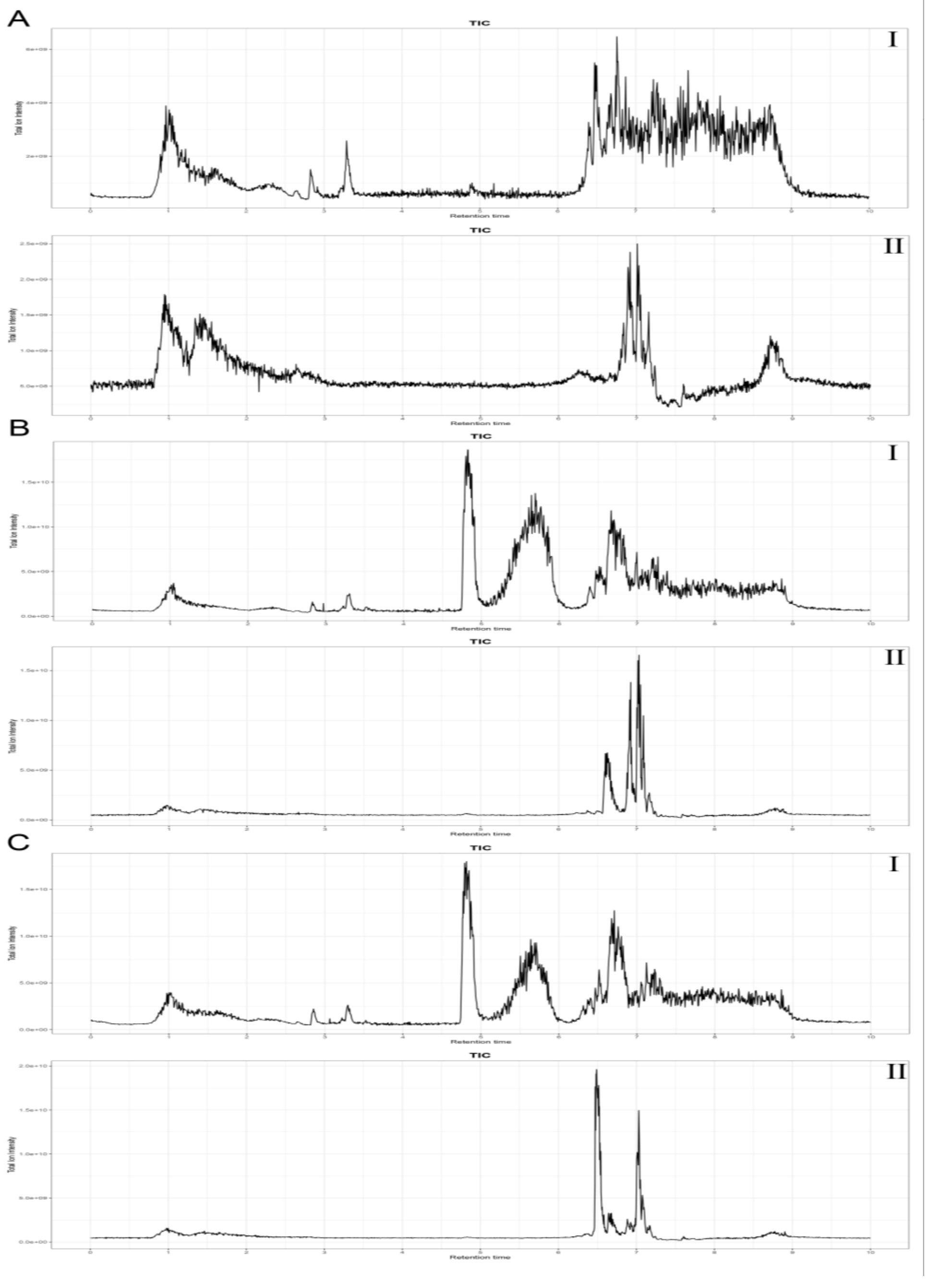

Figure S2. The chemical structures of 161 potential biomarkers.

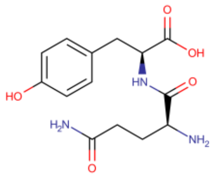

Glutaminyltyrosine

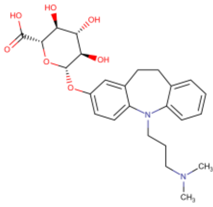

2-Hydroxy-imipramine glucuronide

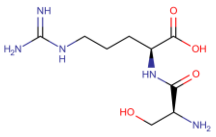

Serylarginine

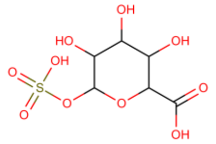

3,4,5-trihydroxy-6-[(2-methylpropa-noyl) oxy]oxane-2-carboxylic acid

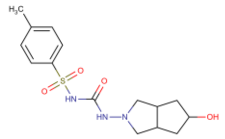

7-Hydroxygliclazide

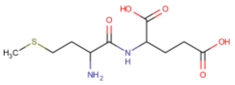

Methionyl-Glutamate

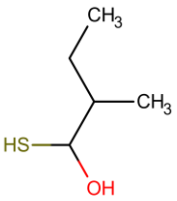

3-Mercapto-2-methyl-1-butanol

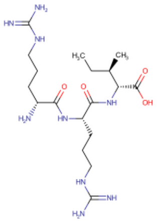

Dynorphin A (6-8)

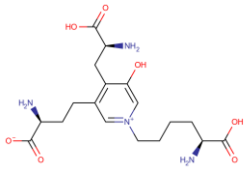

Deoxypyridinoline

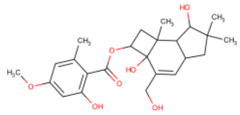

Melleolide B

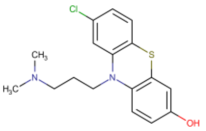

Hydroxychlorpromazine

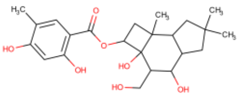

Armillane

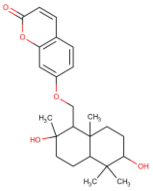

Nevskin

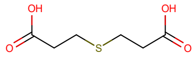

3,3'-Thiobispropanoic acid

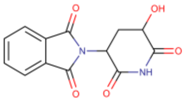

Cis,trans-5'-Hydroxythalidomide

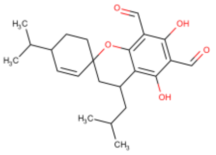

Euglobal IIb

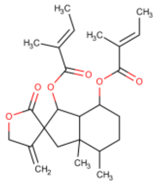

Homofukinolide

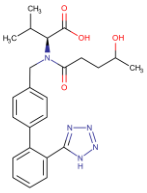

4-Hydroxyvalsartan

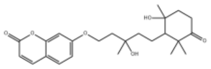

decahydronaphthalen-1-yl) methoxy]-6-hydroxy-2H-chromen-2-one

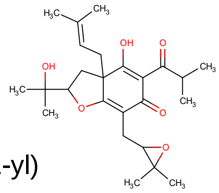

Colupdox a

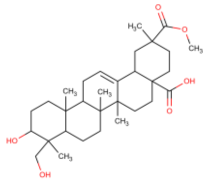

Phytolaccinic acid

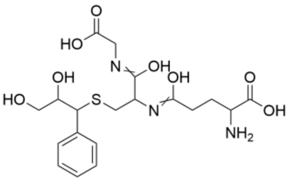

2-amino-4-({1-[(carboxymethyl)-C-hydroxycarbonimidoyl]-2-[(2,3-dihydroxy-1-phenylpropyl)sulfanyl]ethyl}-C-hydroxycarbonimidoyl)butanoic acid

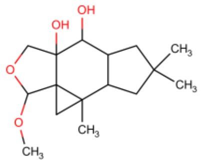

Lactapiperanol C

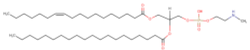

PE(18:0/20:0)

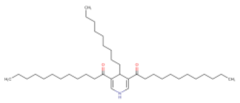

1,1'-(1,4-Dihydro-4-nonyl-3,5 -pyridinediyl)bis[1-dodecanone]

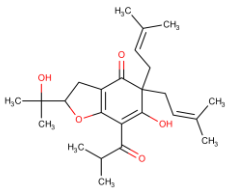

Colupox a

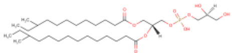

PG(a-13:0/a-15:0)

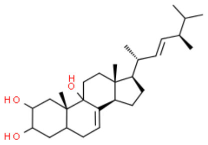

(2β,3α,9α,24R)-Ergosta-7,22-diene-2,3,9-triol

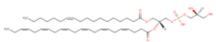

PG(18:1(11Z)/22:6 (4Z,7Z,10Z,13Z,16Z,19Z))

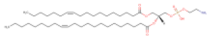

PE(18:1(11Z)/ 22:1(13Z))

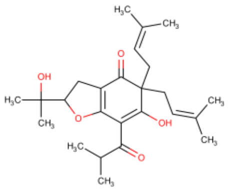

Colupox a

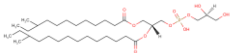

PG(a-13:0/a-15:0)

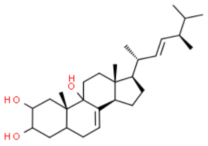

(2β,3α,9α,24R)-Ergosta-7,22-diene-2,3,9-triol

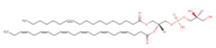

PG(18:1(11Z)/22:6 (4Z,7Z,10Z,13Z,16Z,19Z))

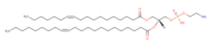

PE(18:1(11Z)/ 22:1(13Z))

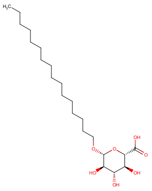

Palmitoyl glucuronide

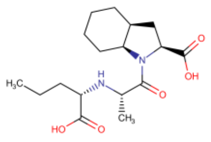

Perindoprilat

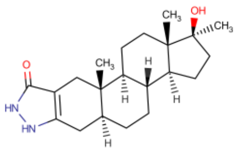

3'-Hydroxystanozolol

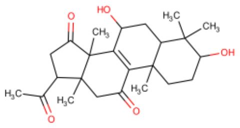

Lucidone A

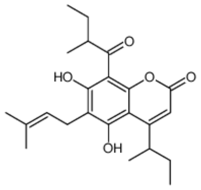

5,7-dihydroxy-4-(1-hydroxypropyl)-6 -(3-methylbut-2-en-1-yl)-8-(2-methylbutanoyl) -2H-chromen-2-one

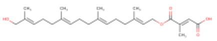

Cavipetin D

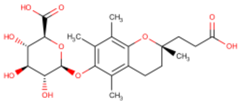

α-CEHC glucuronide

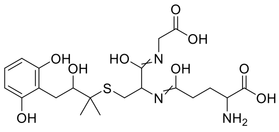

2-amino-4-({1-[(carboxymethyl)-C-hydroxycarbonimidoyl]-2-{[4-(2,6dihydroxyphenyl)-3-hydroxy-2-methylbutan-2-yl]sulfanyl}ethyl}-C-hydroxycarbonimidoyl)butanoic acid

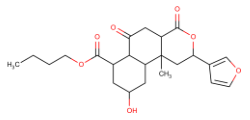

Diosbulbin H

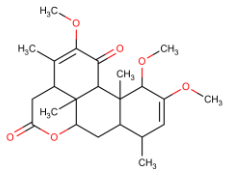

Methylquassin

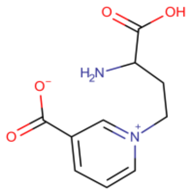

L-Nicotianine

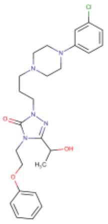

Hydroxynefazodone

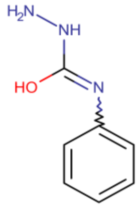

Chondroitin sulfate E (GalNAc4,6diS-GlcA), precursor 5a

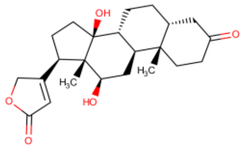

3-keto-Digoxigenin

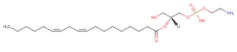

LysoPE (0:0/18:2(9Z,12Z))

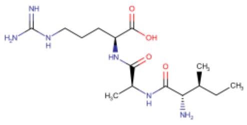

Kinetensin 1-3

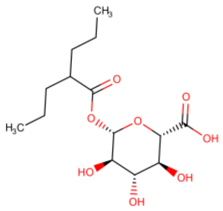

Valproic acid glucuronide

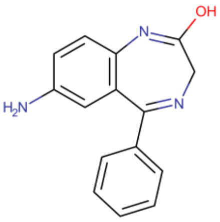

7-Aminonitrazepam

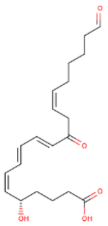

N-Acetyldjenkolic acid

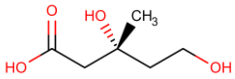

Mevalonic acid

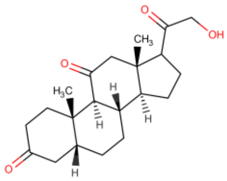

21-Hydroxy-5b-pregnane -3,11,20-trione

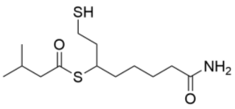

S-(3-Methylbutanoyl) -dihydrolipoamide-E

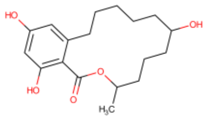

Zeranol

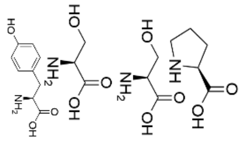

Pro Ser Ser Tyr

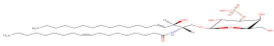

3-O-Sulfogalactosyl -ceramide (d18:1/18:1(9Z)

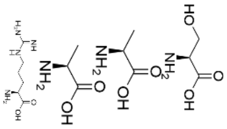

Ser Ala Ala Arg

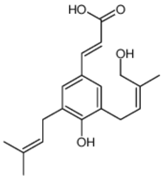

Capillartemisin A

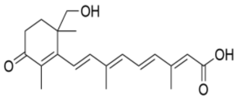

16-Hydroxy-4-oxoretinoic acid

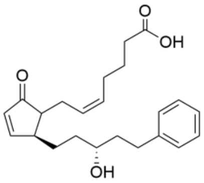

17-phenyl trinor-13,14 -dihydro Prostaglandin A2

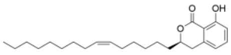

(3R, 6'Z)-3,4-Dihydro-8-hydroxy-3-(6-pentadecenyl)-1H-2-benzopyran-1-one

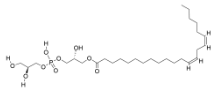

PG(22:2(13Z,16Z)/0:0)

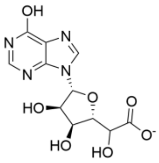

Inosine-5'-carboxylate

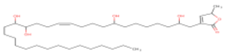

Muricatenol

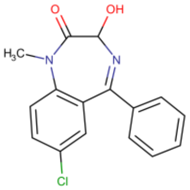

Temazepam

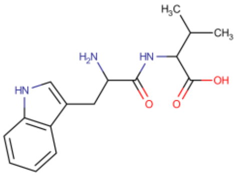

Tryptophyl-Valine

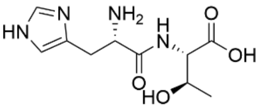

Histidinyl-Threonine

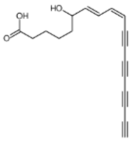

6-hydroxy-7E,9E-Octadecadiene-11,13,15,17-tetraynoic acid

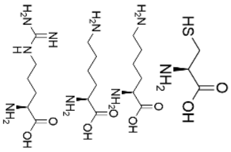

Cys Lys Lys Arg

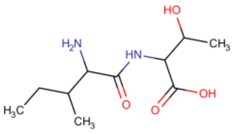

Isoleucyl-Threonine

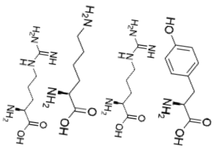

Tyr Arg Lys Arg

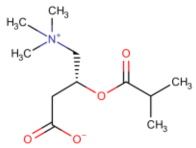

Isobutyrylcarnitine

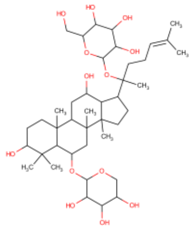

Pseudoginsenoside RT3

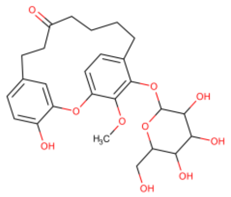

Myricatomentoside I

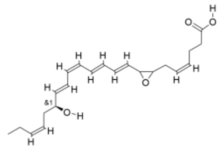

7,8-epoxy-17S-HDHA

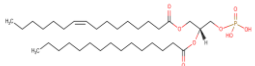

PA(16:1(9Z)/15:0)

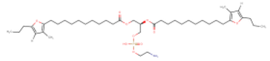

PE(MonoMe(
11,3)/MonoM
e(11,3))

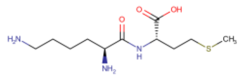

Lysyl-Methionine

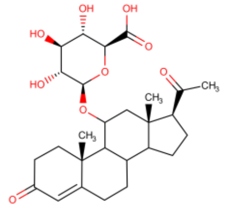

11-Hydroxyprogesterone
11-glucuronide

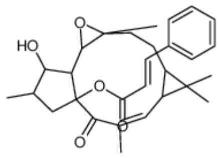

(-)-Jolkinol B

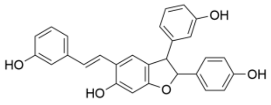

3-(3-hydroxyphenyl)-2-(4-
hydroxyphenyl)-5-[(E)-2-
(3hydroxyphenyl)ethenyl]-
2,3-dihydro-1 benzofuran-6-ol

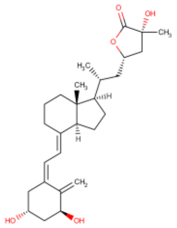

1,25-Dihydroxyvitamin
D3-26,23-lactone

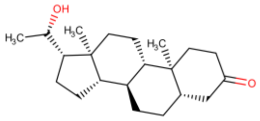

5α-Pregnan-20α-ol-3-one

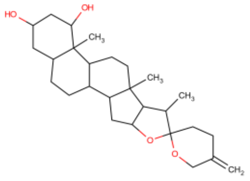

Australigenin

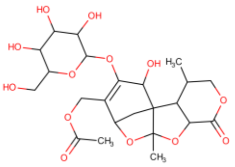

Cyclocalopin D

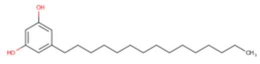

Adipostatin A

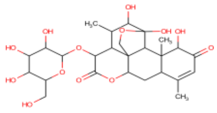

Glaucarubolone 15
-O-β-D-glucopyranoside

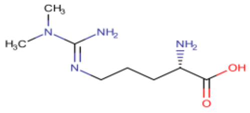

Asymmetric
dimethylarginine

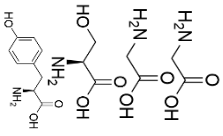

Gly Glu Ser Tyr

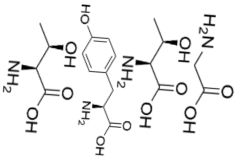

Gly Thr Tyr Thr

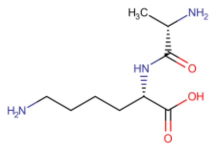

Alanyl-Lysine

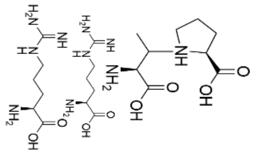

Pro Val Arg Arg

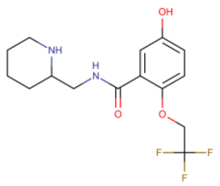

Meta-O-Dealkylated
flecainide

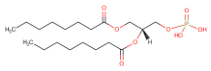

PA(8:0/8:0)

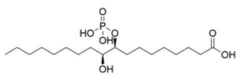

(9S,10S)-10-hydroxy-9-
(phosphonooxy)
octadecanoic acid

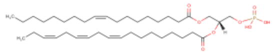

PA(18:1(9Z)/18:3
(9Z,12Z,15Z))

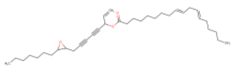

Panaxydol linoleate

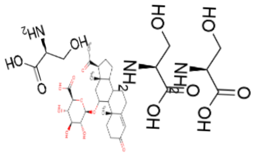

Pro Pro Thr Pro

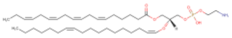

PE(18:4(6Z,9Z,12Z,15Z)/P-18:1(11Z))

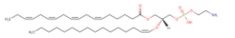

PE(18:4(6Z,9Z,12Z,15Z)/P-16:0)

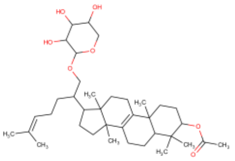

Tsugarioside B

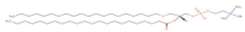

PC(o-20:0/22:0)

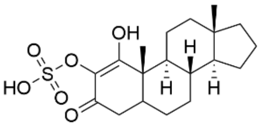

3b,16a-Dihydroxyandrostenone sulfate

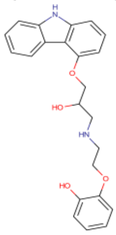

O-Desmethylcarvedilol

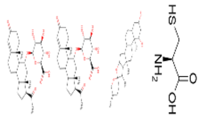

Cys Val Thr Thr

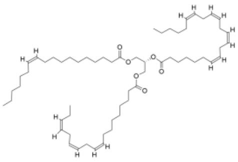

TG(18:1(11Z)/22:4(7Z,10Z,13Z,16Z)/18:3(9Z,12Z,15Z))

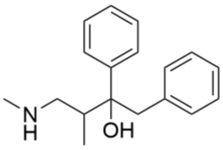

3-methyl-4-(methylamino)-1,2-diphenylbutan-2-ol

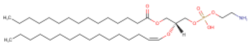

PE(15:0/P-16:0)

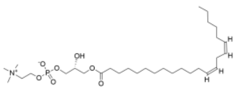

LysoPC(22:2(13Z,16Z))

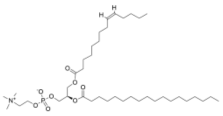

PC(14:1(9Z)/18:0)

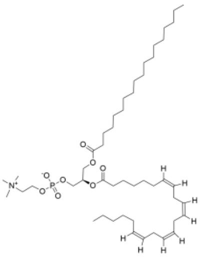

PC(18:0/22:4(7Z,10Z,13Z,16Z))

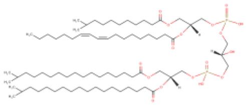

CL(i-12:0/18:2(9Z,11Z)/i-18:0/i-19:0)

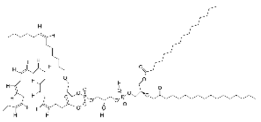

CL(18:0/18:0/16:1(9Z)/22:6(4Z,7Z,10Z,13Z,16Z,19Z))

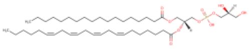

PG(18:0/22:4(7Z,10Z,13Z,16Z))

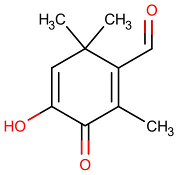

4-Hydroxy-2,6,6-trimethyl-3-oxo-1,4-cyclohexadiene-1-

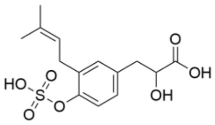

2-hydroxy-3-[3-(3-methylbut-2-en-1-yl)-4-(sulfooxy)phenyl]propanoic acid

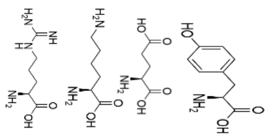

Ser Glu Ser His

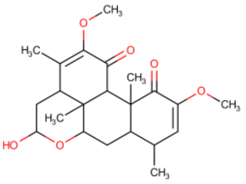

Nigakihemiacetal B

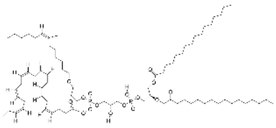

CL(16:0/18:0/18:0/22:5(7Z,10Z,13Z,16Z,19Z))

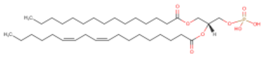

PA(15:0/18:2(9Z,12Z))

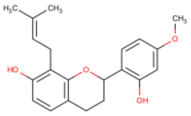

2',7-Dihydroxy-4'-methoxy-8-prenylflavan

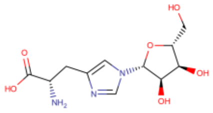

N-Ribosylhistidine

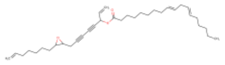

Ginsenoyne A linoleate

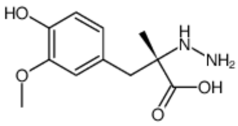

(2S)-2-hydrazinyl-3-(4-hydroxy-3-methoxyphenyl)-2-methylpropanoic acid

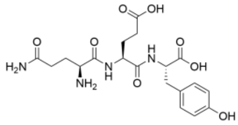

Gln Glu Tyr

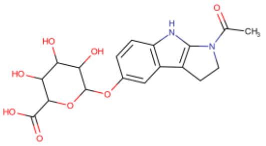

Cyclic N-Acetylserotonin glucuronide

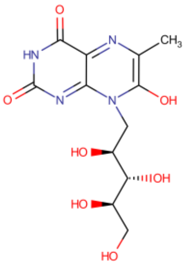

7-Hydroxy-6-methyl-8-ribityl lumazine

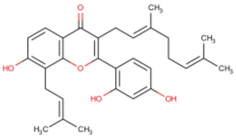

Rubraflavone B

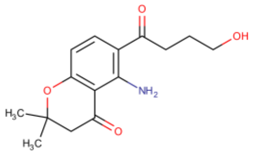

3'-Deaminofusarochromanone

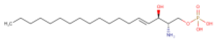

Sphingosine 1-phosphate

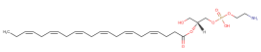

LysoPE(0:0/22:6(4Z,7Z,10Z,13Z,16Z,19Z))

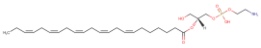

LysoPE(0:0/22:5(7Z,10Z,13Z,16Z,19Z))

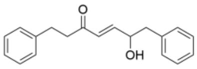

(4E)-6-hydroxy-1,7-diphenylhept-4-en-3-one

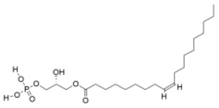

PA(19:1(9Z)/0:0)

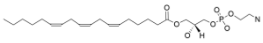

PE(18:3/0:0)

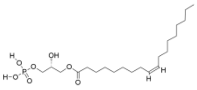

1-Oleoyl Lysophosphatidic Acid (sodium salt)

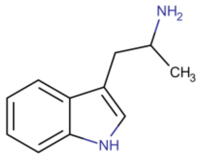

α-Methyltryptamine (AMT)

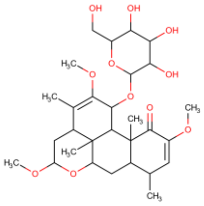

11-α-O-β-D-Glucopyranosyl-16α-O-methylneoquassin

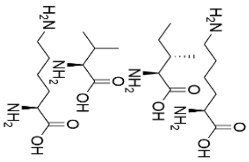

Lys Ile Val Lys

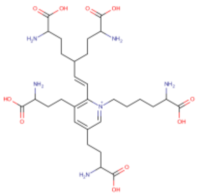

Pentasine

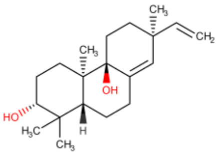

Oryzalexin E

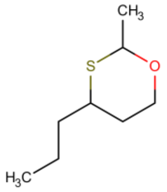

2-Methyl-4-propyl-1,3-oxathiane

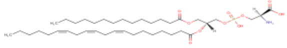

PS(14:0/20:3(8Z,11Z,14Z))

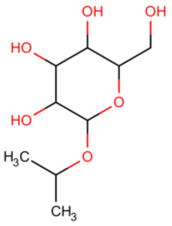

Isopropyl β-D-glucoside

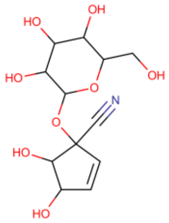

Gynocardin

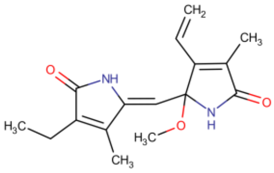

(+/-)-Rollipyrrole

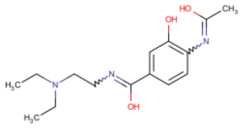

N-Acetyl-3-hydroxyprocainamide

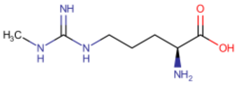

L-Targinine

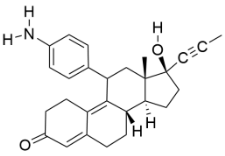

N-Didesmethylmifepristone (RU 42848)

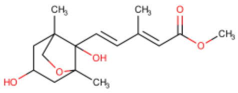

Methyl dihydrophaseate

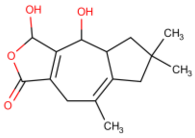

Blennin B

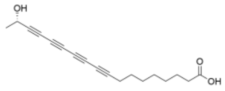

(S)-17-Hydroxy-9,11,13,15-octadecatetraynoic acid

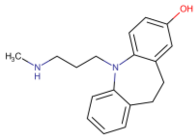

2-hydroxydesipramine

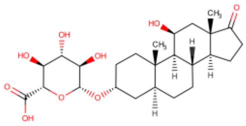

11-β-Hydroxyandrosterone-3-glucuronide

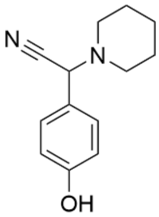

Girgensonine
